# Endless forms most beautiful

**DOI:** 10.4102/sajid.v37i2.388

**Published:** 2022-02-14

**Authors:** Jeremy Nel

**Affiliations:** 1 Division of Infectious Diseases, Helen Joseph Hospital, Johannesburg, South Africa; 2 Division of Infectious Diseases, Department of Medicine, University of the Witwatersrand, Johannesburg, South Africa

It is an unsettling thing to be asked to write a commentary on your own career, especially when you feel it’s barely begun! You can add to that the somewhat awkward problem of being surrounded by actual luminaries in the Infectious Diseases field in South Africa whose names bookend author lists in the *New England Journal of Medicine* with embarrassing regularity. And then there’s the imprecision from which all self-reflection inevitably suffers. That being said, here follows my best attempt.

I currently head up the Division of Infectious Diseases at Helen Joseph Hospital and the University of the Witwatersrand, and I came to the field in an unusual way. Starting towards the end of my undergraduate training and proceeding right through registrar time, I developed a keen fascination (an obsession, if I’m honest) with evolutionary theory. With its logic animated initially by the numinous beauty of Richard Dawkins’s writing, I became enamoured with the natural world and enthralled by how the physics of evolution shaped it. Popular science books became textbooks, and then university courses and my head became filled with population genetics, selection coefficients, linkage disequilibria, and the like. Realising at some level that I risked turning into a frustrated biologist, I gravitated towards the one branch of internal medicine that routinely looked beyond *Homo sapiens* and at least acknowledged a few tens of thousands of other organisms too. Each of these evolutionary compatriots has evolved for just as long as we have, and so discovering them one after another feels like entering a cave of wonders every time. Who could ever have thought that *Toxoplasmosa gondii* renders its rodent host fearless in the face of cats, ensuring the hapless creature is eaten and thereby completing the parasite’s lifecycle? Or that the ancestors of trypanosomes were abruptly forced into South American and African lineages when the two continents split apart over 100 million years ago? Or that almost every inherited disorder of haemoglobin that you can name, from sickle cell disease to thalassaemia and back, represents an evolutionary ‘scar’ in the genome from our battle with malaria, a pathogen responsible for more human deaths in history than any flood, plague, or axe?

Overflowing with the excitement of these discoveries, I knew I would never get bored in the field of Infectious Diseases. What I then needed was to find a way to study it. Here I had the tremendous good fortune of finding two people as my academic godfathers. Dave Spencer, the doyen of HIV medicine in the region, had just come in to run Infectious Diseases at Helen Joseph Hospital. He was soon joined by Chris Lippincott, himself fresh from the University of North Carolina and arriving in Johannesburg determined to pull infectious diseases in Johannesburg up by its bootstraps. I can’t begin to do justice to the mentoring role they played professionally and personally; doubtless, I would have ended up in some or other ditch professionally without them at the wheel occasionally. From them, I learnt the imperative of mentoring in academia and have tried to recapitulate this, in turn, with the fellows I have helped to train. I count both Dave and Chris among my close friends to this day.

My academic career isn’t far enough for me to really make sense of. Still, I have been fortunate with the number of opportunities that have come my way, many of which I still feel absurdly undeserving of. At times (including right now!), there has been so much on offer that I’ve felt drawn to that and had stretched myself a bit too thinly across projects. That’s probably something that I’ll need to get better at managing. However, if some minor advice is not too presumptuous, I think that I would still encourage junior colleagues to follow the counsel of another mentor – Prof Mike Cohen, who once sanguinely advised, ‘If a door opens, walk through it!’ In this field, there really are more possibilities than we can imagine.

I still find it wondrous that Infectious Diseases is capacious enough for anyone to undertake any one of dozens of satisfying and separate careers within it. HIV medicine, transplant infectious diseases, antimicrobial stewardship and myriad other possibilities are all open to anyone interested. One’s research interests can span any assortment of thousands of infections (I will never understand how my colleagues in other internal medicine subspecialties manage to beat back boredom staring at a single organ for 30 years but to each their own …). Combine that with the great need for infectious diseases specialists in sub-Saharan Africa, and I doubt that there is another speciality that offers a greater chance for a fulfilling career. I have never wondered why I get up in the morning, and I would like to think that, over time, that might add up to something meaningful.

